# An efficient static sampling method for in situ measurement of rhizosphere volatile organic compounds in plant–soil systems

**DOI:** 10.1186/s13007-026-01573-y

**Published:** 2026-07-27

**Authors:** Anne-Sophie Wachter, Monica Barman, Nicole M. van Dam, Mika Tarkka, Doris Vetterlein

**Affiliations:** 1 Helmholtz Centre for Environmental Research–UFZ, Theodor-Lieser-Str. 4, 06120 Halle (Saale), Germany; 2 Leibniz Institute of Vegetable and Ornamental Crops–IGZ, Theodor-Echtermeyer-Weg 1, 14979 Großbeeren, Germany; 3 Institute for Biodiversity, Ecology and Evolution-IBEE, Friedrich Schiller University Jena, Dornburgerstraße 159, 07743 Jena, Germany

**Keywords:** Root VOCs, Static sampling, Silicone adsorbents, *Malus domestica*, Rhizosphere, Plant–soil interactions, Gas chromatography/mass spectrometry, Terpenoids

## Abstract

**Background:**

Volatile organic compounds (VOCs) play a crucial role in mediating the interactions of plants with their environment. Research has predominantly addressed aboveground VOCs, whereas belowground emissions-especially in undisturbed plant–soil systems—remain largely unexplored. Our study aimed to develop a method for the in situ trapping of rhizosphere VOCs using a static sampling approach. Apple (*Malus domestica* Borkh.) plantlets were grown in soil-filled, perforated rhizoboxes. The holes were integrated to insert silicone-based polymeric phases (Sorb-Stars^®^) for the collection of VOCs. Sampling was repeated four times at one-week intervals. As VOCs found in the rhizosphere may derive from multiple origins, additional trapping setups were employed to obtain emissions specifically from roots (hydroponic-grown plants), shoots (aboveground VOC collection), and soil (soil-only rhizoboxes).

**Results:**

Using thermal desorption followed by gas chromatography/ mass spectrometry (GC/MS), a total of twelve VOCs were detected in the rhizosphere samples. Seven of these were successfully identified: 2-furanmethanol, longifolene, 2-(2-hydroxypropoxy)-1-propanol, α-dihydroterpineol, α-terpineol, 3(2H)-benzofuranone, and butylated hydroxytoluene. All of these have been documented in previous studies as VOCs emitted in biological systems. The other five VOCs could not be annotated. Six of the detected VOCs could be classified as the “core volatilome”, being present in all trapping setups, while the others showed more setup-specific occurrence. Notably, 3(2H)-benzofuranone was the only VOC detected exclusively in the rhizosphere samples, highlighting the necessity of plant–soil interactions for its production. The setup and sampling time at which the highest relative abundance was observed varied considerably between compounds, emphasizing the dynamic nature of belowground VOC emissions. Therefore, an optimal sampling time could not be identified.

**Conclusions:**

We demonstrated that the proposed method offers a suitable approach to non-invasively capture VOCs in the rhizosphere of plant–soil systems. It was further shown that additional setups, focusing on isolated below- and aboveground components, may help determine the VOC emitters.

**Supplementary Information:**

The online version contains supplementary material available at 10.1186/s13007-026-01573-y.

## Background

Volatile organic compounds (VOCs) are secondary metabolites emitted by all forms of life ([1], and citations therein). The physicochemical constraints on volatility limit VOCs to low-molecular and largely lipophilic products, including terpenes, sulphur- and nitrogen-containing compounds, fatty acid derivatives, and aromatic compounds [1–4].

In plants, VOCs are important infochemicals, as they play a crucial role in the interaction with their environment. They are an integral part of how plants adapt to their environment and communicate amongst themselves and other living organisms [2, 3, 5]. In addition, VOCs facilitate within-plant signaling, enabling communication between different organs, such as roots and shoots [3].

Research on plant VOCs has focused mainly on shoot VOCs and aboveground interactions, while the complex networks of volatile-mediated communication belowground have received far less attention [2, 3, 6, 7]. So far, studies have shown that VOCs emitted by roots and also soil microbes are integral components of the soil food web. They mediate processes such as plant protection against root herbivores and pathogens, inhibition of neighboring plant growth, and the establishment of beneficial symbioses [1, 8, 9].

The limited research on belowground VOC dynamics can largely be attributed to the technical challenges associated with sampling volatiles in undisturbed plant–soil systems. To reduce experimental complexity, soil is often excluded from setups, with plants grown in hydroponic or aeroponic systems (e.g., [10, 11]). Alternatively, VOCs are measured only after the roots have been washed (e.g., [12, 13]). However, studies focusing on isolated components may not reflect the full complexity of belowground communication, which limits their transferability to undisturbed environments. Also, physically disturbing the roots before sampling (e.g., through root washing) may alter the volatile profile, since plants emit different compounds in response to tissue damage [14, 15].

The aim of this work was to develop a method for the in situ measurement of VOCs in plant–soil systems. Specifically, we targeted the soil zone directly influenced by root-emitted VOCs, which is hereafter referred to as the rhizosphere. Dynamic and static headspace techniques coupled with gas chromatography/mass spectrometry (GC/MS) have become the standard methods for measuring belowground volatile emissions [1]. In dynamic headspace methods, an active continuous airflow passes through the sampling container and carries VOCs to an adsorbent matrix for collection (e.g., [4]). This results in a composite volatile profile representing the entire container, meaning selective sampling of the rhizosphere is not possible. Moreover, the airflow can disturb the system, potentially altering the volatile emission patterns [16].

In contrast, VOCs can be passively trapped onto an adsorbent, without the need for air flow. The standard approach for this static headspace technique is solid-phase microextraction (SPME), which uses an adsorbent-coated fused silica fiber to collect VOCs [1]. Other silicon-based polymeric sorbents with larger active surfaces are also commonly used to improve compound adsorption, such as polydimethylsiloxane (PDMS) tubing or stir-bar sorptive extraction (SBSE) [1, 16].

Weidenhamer et al. [17] designed a rhizobox-based method to monitor allelochemical dynamics through non-destructive VOC sampling in the vicinity of roots. In this setup, holes were drilled at regular intervals through the rhizoboxes. PDMS tubes were wrapped around empty syringes for structural support and then inserted into the holes. To collect the VOCs, a solvent was passed through the PDMS tubes, and the resulting extract was analyzed using high-performance liquid chromatography (HPLC). This method successfully enabled the detection of thiophenes exuded by *Tagetes patula* roots, allowing their spatial and temporal dynamics to be tracked effectively belowground.

The setup described in our study was based on this rhizobox approach introduced by Weidenhamer et al. [17]. Instead of *Tagetes patula* apple (*Malus domestica* Borkh.) plantlets were selected as the experimental species. As a major fruit crop of global economic relevance, deciphering its signaling mechanisms is of considerable interest. The feasibility of capturing belowground apple VOCs has been shown by Abraham et al. [18], who monitored herbivore-induced emissions using a dynamic sampling system.

Unlike the Weidenhamer et al. [17] method, we employed GC/MS coupled with a thermal desorption unit for compound recovery. The reason for this change was that thermal desorption (TD) offers several advantages over solvent extraction. These include higher desorption efficiency, the prevention of sample dilution, and the elimination of potential impurities introduced by solvents [4].

Sorb-Stars^®^ were employed to capture VOCs. These silicon-based polymeric adsorbents were developed to be highly sorptive towards organic compounds with medium volatility [19]. They have been applied in various contexts, including forensic investigations [20] and monitoring the migration of mineral oil from packaging into food [19]. While they have rarely been used for capturing plant VOCs, Zytowski & Baldermann [21] recently expanded their application by demonstrating that Sorb-Stars^®^ can be utilized to capture polystyrene nanoparticles in pak choi *(Brassica rapa* subsp*. chinensis)*.

Targeted sampling of the rhizosphere introduces the challenge of attributing the detected VOCs to their origin. The rhizosphere is a highly complex and heterogeneous microenvironment in which VOCs may arise from multiple sources. Detected VOCs can originate from the soil microbiome or the roots themselves [22], or may be the result of their specific interaction. Furthermore, shoot-derived VOCs may be transported to root tissues and consequently appear belowground. To address this complexity, we complemented our belowground VOC sampling in plant–soil rhizoboxes with additional trapping setups. Specifically, we used (i) soil-only rhizoboxes and (ii) hydroponically grown plants to distinguish between soil- and root-derived VOCs and to assess plant–soil interaction effects, and (iii) aboveground VOCs to investigate potential root-to-shoot transport.

To capture temporal variation in VOC dynamics, we inserted adsorbents four times at one-week intervals. This was done to identify the period of highest volatile emission and thus the optimal sampling time.

In summary, our aim was to introduce a novel setup for static VOC collection in undisturbed plant–soil systems, addressing the following questions: (1) Was the proposed method efficient, i.e., capable of capturing VOCs in the rhizosphere? (2) Could the origins of the VOCs be traced using additional trapping setups? (3) Did the volatile emission patterns indicate an optimal time frame for sampling?

## Methods

### Experimental design

Three different experimental systems were established: plants grown in rhizoboxes with soil, soil-only rhizoboxes without plants, and plants grown in hydroponics. Each system included four replicates, with “replicate” referring to an individual rhizobox or hydroponic pot. For the plant-containing systems, two plants were grown per rhizobox or pot.

### Plant material and substrate

The experiment was conducted with apple (*Malus domestica*) rootstock genotype M26, which was propagated in vitro as described by Rohr et al. [23]. 15-week-old plantlets were used for the experiment. In August 2024, one month before the experiment, topsoil (0–20 cm) was collected from the experimental field site at Ellerhoop, Germany (53.71435° N, 9.770143° E). The soil was a loamy sand classified as an Endostagnic Luvisol [24]. Additional soil characteristics were provided in Mahnkopp et al. [25].

### Rhizobox setup

**Fig. 1:**
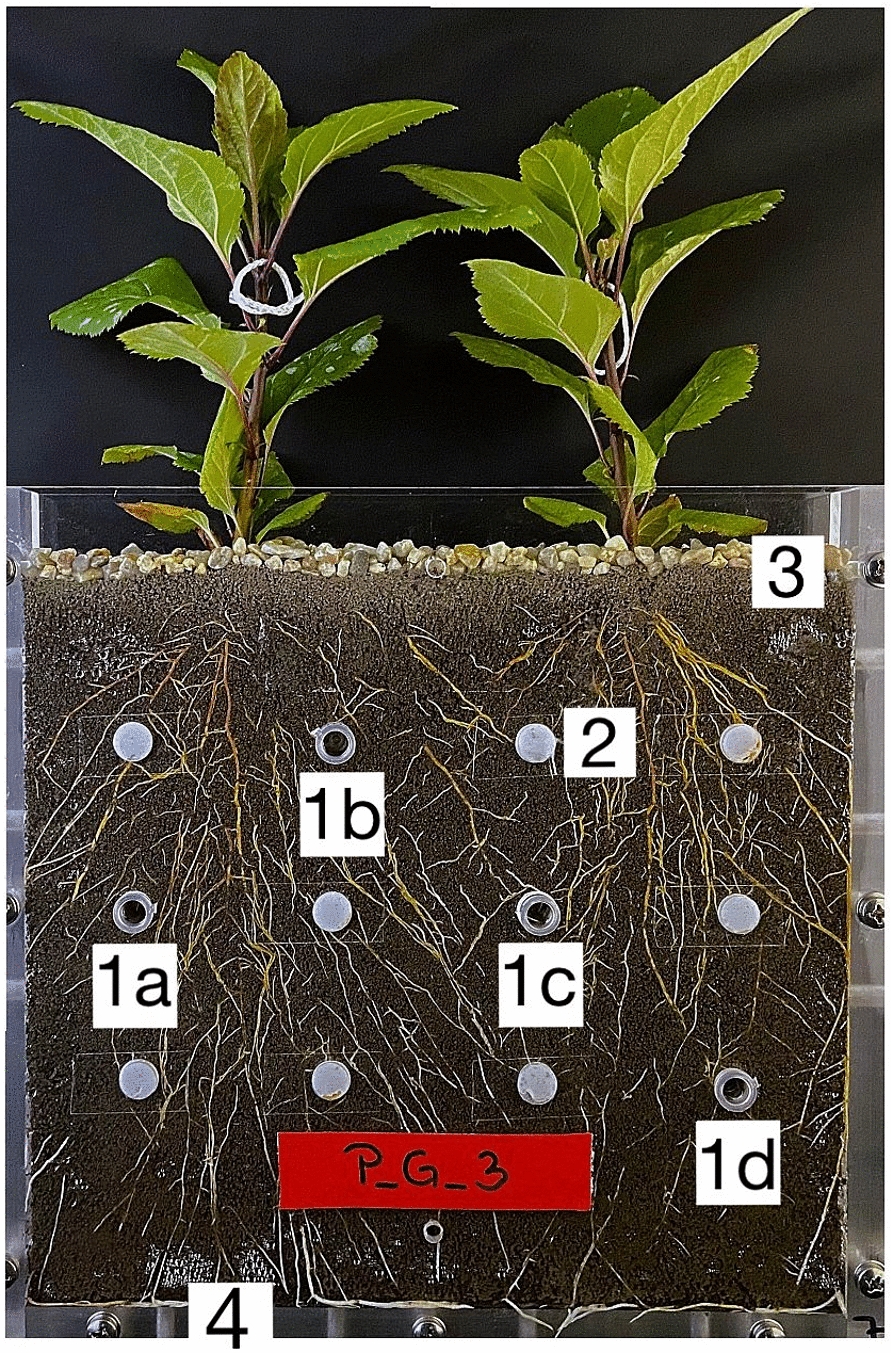
Rhizobox with apple plantlets and soil showing one representative replicate 34 days after planting. The labels indicate: **1** (a–d) sampling ports (sampled holes containing the perforated microcentrifuge tubes), **2** non-sampled holes sealed with tape to retain the substrate, **3** a layer of coarse gravel to reduce surface evaporation, and **4** irrigation holes in the bottom of the rhizobox (four holes with Ø 4 mm). White strings mark the youngest fully expanded leaf at the start of the experiment. Note that all roots visible through the Plexiglas^®^ front plate developed during the experiment

Rhizoboxes (inner dimensions 20.5 × 20.5 × 2.2 cm, outer dimensions 22.5 × 21.5 × 3 cm; height × width × depth) were constructed from Plexiglas^®^ that contained twelve evenly spaced holes (Ø 0.9 mm) in the front and back plates (Fig. 1). These sampling ports were arranged at three depths (5.8 cm, 10.1 cm, and 14.4 cm), with four ports per depth.

To ensure homogeneous distribution, the front plate was removed during filling, and the substrate was added while the rhizoboxes were positioned horizontally with their back plate facing downward. Rhizoboxes were then filled to a height of 19.5 cm with 2 mm-sieved substrate (bulk density 1.1 g cm^−3^). In the rhizoboxes containing plants, apple plantlets were placed on top of the soil once half of the substrate had been added.

A stiff Tygon^®^ tube (7 mm outer Ø), sealed at one end with tape, was used to push the substrate out of the sampling ports, after which a modified microcentrifuge tube (measuring jar 30.9.0010, GONOTEC GmbH, Berlin; 0.7 cm inner Ø, 3 cm length) was inserted.

The tubes were introduced to prevent direct contact between soil and the adsorbents, while they were perforated to ensure a continuous passage of VOCs. For perforation, the lid of the tubes was removed, and nine holes (3–4 mm in diameter) were drilled along the tube wall. The first perforation was positioned ~ 4 mm from the opening, followed by two additional perforations spaced 2–3 mm apart. The remaining six holes were arranged in two groups of three, evenly distributed around the tube (Fig. S1).

### Hydroponic setup

For the hydroponic system, plantlets were inserted into foam-plugged holes within a perforated disk that was placed on 700 mL plastic pots. The pots were filled to two-thirds capacity with half-strength Hoagland solution, adjusted for apple as described by Chang et al. [26]. This solution comprised 2.5 mmol L^−1^ Ca (NO_3_)_2_, 1 mmol L^−1^ MgSO_4_, 0.5 mmol L^−1^ (NH_4_)H_2_PO_4_, 3 mmol L^−1^ K_2_SO_4_, 0.1 mmol L^−1^ Fe-Na-EDTA, 0.2 µmol L^−1^ CuSO_4_, 1 µmol L^−1^ ZnSO_4_, 20 µmol L^−1^ H_3_BO_3_, 0.05 µmol L^−1^ (NH_4_)_6_Mo_7_O_24_, and 1 µmol L^−1^ MnSO_4_. The pH was adjusted to 6.5 using 1 M HCl. Continuous aeration was provided using an air pump. The nutrient solution was changed twice weekly, increasing to three times per week starting on day 27.

### Growth conditions

Rhizoboxes were placed on melamine trays and wrapped in aluminum foil to prevent algal growth. They were watered from top and bottom to a volumetric water content of 22%, which was maintained throughout the experiment by watering twice every week.

The growth chamber was maintained at 22 °C during the day and 18 °C at night, with a 16 h light-period and a constant relative humidity of 70%. Photosynthetically active radiation was initially set to 150 µM m^−2^ s^−1^ to prevent leaf photodamage and increased to 260 µM m^−2^ s^−1^ after an adjustment period on day 9 after planting. Harvest was conducted on day 37 after planting.

### Preparation of adsorbents

For VOC collection, an adsorbent made out of a silicon-based polymeric phase was used, which is marketed under the registered trademark Sorb-Star^®^ (ENVEA, Poissy, France). Sorb-Stars^®^ (cylinders with 2 cm length and Ø 2 mm) were obtained from Avantor (Radnor, PA, USA). Prior to the experiment, the adsorbents were tested for their ability to capture a mixture of synthetic standard VOCs with known concentrations. As shown in Fig. S2, the adsorbents showed satisfactory adsorption performance for all the standards used.

They were cleaned by heating at 240 °C for 60 min under a constant nitrogen flow of 5 L min^−1^ in a nitrogen-purged oven. When extracting the oven vials, they were sealed with Teflon^®^ tape at both ends to prevent exchange with ambient air.

After this step, the adsorbents were only handled with tweezers that were cleaned with HPLC-grade n-hexane prior to use. Under a fume hood, the adsorbents were transferred into glass containers, and Teflon^®^ tape was wrapped around the lids. These containers had been oven-cleaned beforehand at 200 °C for 30 min.

To prepare for VOC collection, the cleaned adsorbents were placed individually into 1.5 mL glass vials under a fume hood. The vials were then fitted with screw caps and sealed with Parafilm^®^ until used for volatile collection.

### VOC trapping setups

VOCs were collected in four different trapping setups, as summarized in Table 1.

**Table 1:** Overview of the VOC trapping setups

Illustration	Origin	System	Location	Function
	Rhizosphere	Rhizobox with plants and soil	Belowground	VOC detection and their analyses
	Shoot	Rhizobox with plants and soil	Aboveground	Analyzed for VOCs found in the rhizosphere, to track their origins
	Soil	Rhizobox with only soil	Belowground	
	Root	Hydroponically grown plants	Belowground	

Colored boxes indicate which part of the setup was sampled

**Fig. 2:**
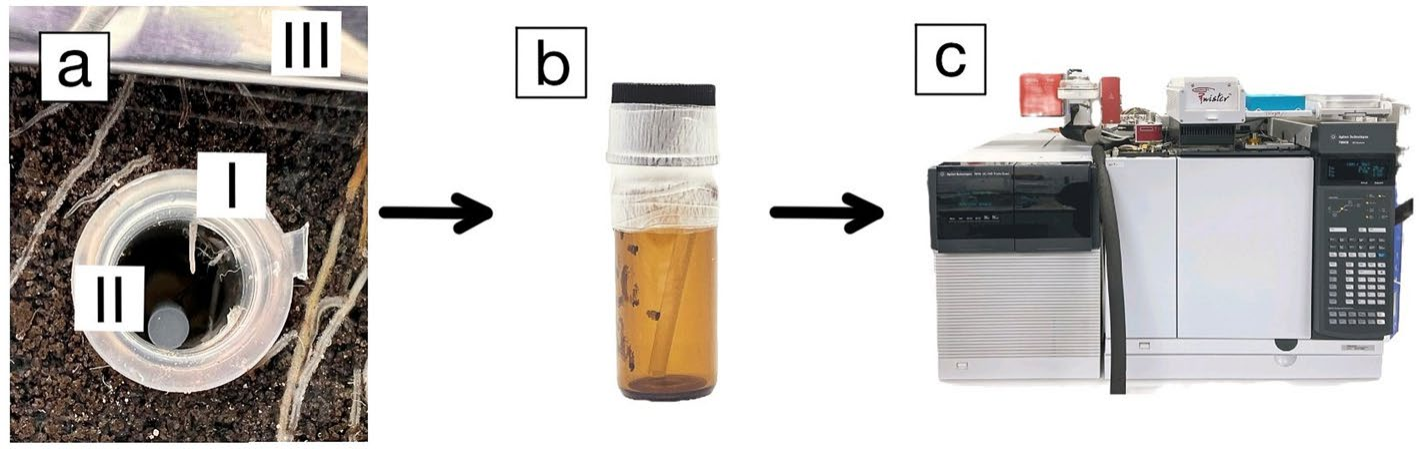
Workflow of sample collection. **a** Adsorbent inserted into a sampling port depicting one representative section of a rhizobox with plant and soil, where I. shows the perforated microcentrifuge tube with a root growing in just below “I”, II. the Sorb-Star^®^, and III. aluminum foil placed over the sampling port during VOC collection; **b** storage of Sorb-Stars^®^ in glass vials wrapped with Parafilm^®^ at − 18 °C; **c** measurement using TD-GC/MS

For the rhizoboxes with plant and soil, four of the twelve holes were used for VOC trapping (labeled a–d in Fig. 1). After inserting the Sorb-Stars^®^, additional aluminum foil was placed over each sampling port and secured with tape at the corners to restrict airflow. VOCs were collected for 24 h, beginning 2 h into the light period (Fig. 2a). VOCs were trapped at weeks 2, 3, 4, and 5 after planting.

The same procedure was applied to the rhizoboxes with only soil.

For the hydroponically grown plants, the collection was performed within the same time frame and frequency. The Sorb-Stars^®^ were pierced with hexane-cleaned wires, the free ends of which were wrapped around the perforated disks supporting the plants. This suspended the Sorb-Stars^®^ in the space between the disk and the nutrient solution. With this configuration, direct contact between the adsorbents and the nutrient solution, the disks, and the plant roots was prevented (Fig. S3). Per pot, two adsorbents were installed at each time point.

Aboveground VOCs were sampled once, at week 4 after planting. For this collection, hexane-cleaned wires were again inserted through the Sorb-Stars^®^, with the free ends positioned in the rhizobox substrate. The adsorbents were thus placed adjacent to the plants while avoiding direct contact with stems or leaves. One adsorbent was positioned per plant. Individual plants were then carefully enclosed in polyester bags (Toppits^®^ “Bratschlauch”, Melitta, Minden, Germany) that were loosely tied with a cotton string (Fig. S4).

Aboveground sampling was initiated after belowground collection to ensure that the potential leaf damage during setup did not affect the belowground measurements from the same sampling event. Therefore, during the third sampling, belowground VOCs were trapped for 24 h, after which the 4 h aboveground collection was conducted.

After trapping, adsorbents were returned to the 1.5 mL glass vials, sealed with Parafilm, and stored at − 18 °C until GC/MS analysis (Fig. 2b).

### GC/MS methods

The collected VOCs were analyzed by TD-GC/MS (Fig. 2c). Collected adsorbents were introduced into the GC system using Gerstel’s (Mühlheim an der Ruhr, Germany) MPS2 multipurpose sampler, Thermal Desorption Unit (TDU), and Cooled Injection System (CIS). Analytical sensitivity was optimized to manage the high abundance of siloxane-related compounds, with the best performance obtained using a purge flow to the split vent of 5 mL/min at 0.01 min in the CIS and a splitless TDU configuration. Desorption with TDU was carried out with the following temperature program: a starting temperature of 25 °C, an increase of 60 °C/min–220 °C, and holding for 8 min. Cryofocusing was performed in the CIS through cryo-cooling at − 50 °C. The temperature was then ramped at 12 °C/s–230 °C and held for 3 min to transfer the analytes onto the GC column.

Separation and detection of compounds were carried out on an Agilent 7890B gas chromatograph coupled to an Agilent 7010 Triple Quadrupole mass spectrometer (Agilent Technologies, Santa Clara, CA, USA). VOCs were separated on a DB-5MS fused silica column (30 m length × 0.25 mm inner Ø, 0.25 µm film thickness) purchased from Agilent Technologies (Santa Clara, CA, USA). Helium was used as a carrier gas maintained at a constant flow rate of 1 mL min^−1^. The GC oven temperature program was adapted from Barman et al. [27] with minor modifications: initial temperature 40 °C, ramped at 6 °C min⁻^1^ to 250 °C (held for 1 min), followed by an additional ramp of 15 °C min⁻^1^ to 280 °C with a final hold of 1 min to facilitate complete elution.

The MS analysis was carried out in full-scan mode with a mass scanning range of *m/z* 35–350. The electron impact ionization energy was 70 eV for all measurements. The source temperature was set to 230 °C, and the quadrupole temperature to 150 °C.

To ensure comparability of peak areas, instrument sensitivity and MS tuning parameters were kept consistent throughout the analytical sequence. This was verified by repeated analysis of known amounts of standard mixtures after every 40 samples.

### Rhizosphere VOC detection and elimination of contaminants

VOCs were searched exclusively for the origin we labeled “Rhizosphere”, which refers to the adsorbents collected from the belowground sampling of the plant–soil rhizoboxes (Table 1). A total of 64 samples were collected in this trapping setup (4 positions within each rhizobox × 4 rhizoboxes × 4 time points). Spectral data were only generated for 61 samples, as three were used for optimizing the GC/MS method. All steps regarding VOC detection, identification, and elimination of contaminants were carried out with Agilent’s MassHunter Workstation Software for Qualitative Analysis (version 10.0, Agilent Technologies). The first step in the chromatogram analysis workflow (Fig. S5) was the selection of chromatograms intended for volatile profiling (i.e., finding VOCs present). For each time point, a subset of chromatograms (evenly distributed across rhizoboxes and positions) was examined in detail, and 2 representative chromatograms were chosen. This resulted in a total of 8 chromatograms used for volatile profiling. These chromatograms were processed (i.e., generate tables of retention times (RT) and peak areas for all peaks), with a filter applied to include only peaks with an area larger than 5000 counts. Each detected peak was then assessed individually to determine whether it represents a VOC or a contaminant, following a defined set of decision criteria.

As a first criterion, sample chromatograms were compared with those of blank Sorb-Stars^®^. Here, “blank” refers to cleaned sorbents that were sealed right away and exposed to ambient air as little as possible before insertion. They therefore represent sorbent- or instrument-derived contaminants. Peaks in the samples matching the RT of blank peaks were considered contaminants.

In the next evaluation step, we assessed whether the spectral data (*m/z*) of a peak were novel. A true VOC should show a distinct and characteristic *m/z*. Therefore, if the *m/z* of the baseline immediately before the peak was identical to that of the peak, it was not considered to be a VOC.

If none of these conditions were met, the peak's spectral data were searched in the NIST 20 mass spectral library (National Institute of Standards and Technology, Gaithersburg, MD, USA) using the NIST Mass Spectral Search Program (version 2.4). If the top library match was a siloxane, the peak was considered a contaminant. Likewise, if *m/z* 207 appeared among the five most abundant ions, the peak was classified as a contaminant, as this ion is characteristic of column-bleed–related impurities (e.g., [28]). Peaks that did not meet any of these criteria for contaminants were considered VOCs.

### Rhizosphere VOC identification and semi-quantification

To identify VOCs, the suggested matches provided by the NIST Mass Spectral Search Program for each peak’s mass spectrum were examined. If at least three of the five most abundant *m/z* values matched, the suggested name was considered. For these candidate VOCs, the Kovats retention index (RI) was researched. The RI of each measured VOC was calculated using alkane standards (C_8_–C_20_) run with TD-GC/MS alongside the samples. The calculated RI was then compared with the literature RI, and the VOC was considered identified if the difference was within ± 50 units. If none of the suggestions matched, the VOC was labeled “UNK” (Unknown).

Then, we built a custom library based on mass spectra and RIs of the detected VOCs using MassHunter Workstation’s Library Editor for Quantitative Analysis (version 10.0, Agilent Technologies), as described in Barman et al. [27]. Each library entry comprised the mass spectrum, with identified VOCs additionally annotated with compound name, Chemical Abstracts Service (CAS) number, and molecular weight.

The custom library was used to detect VOCs in all rhizosphere samples semi-automatically by matching the samples' peak mass spectra to those in the library.

These VOC assignments were verified by retaining only entries with an RT no more than ± 0.1 min away from the value noted during profiling. Peaks were also included if the match score exceeded 80, except if the same VOC appeared multiple times. Only entries with an RT difference of < 1 min and a score > 80 were retained.

### VOC analysis in soil, root, and shoot trapping setups

The generated library was applied not only to the rhizosphere samples, but also to the complementary trapping setups designed to track the origin of the VOCs found in the rhizosphere (Table 1). After the (semi)automatic detection of the VOCs, the same verification steps as for the rhizosphere samples were applied. Overall, 64 soil, 31 root, and 8 shoot chromatograms were analyzed. One root sample was excluded as the recording stopped at RT 9 min.

### Data analysis and visualization

All steps regarding data analysis and visualization were conducted with R (R 4.4.0), [29] in RStudio (version 2023.03.1).

The percentage of contaminants in a chromatogram was calculated as the ratio of peaks assigned as VOCs to the total number of peaks. The eight subset chromatograms were used as representatives to determine the range of contaminant percentages.

For each sample, all peaks assigned to a given VOC were added up to obtain the total area per VOC. These values were compiled into a table (Table S2) that served as the basis for all subsequent data explorations. A VENN diagram was plotted using the package ggVennDiagram (version 1.5.4) [30]. To generate the heatmap, GC/MS-derived peak areas were normalized by adding up the individual samples within each group (trapping setup × sampling time) per VOC. Then, for each VOC, the total across all groups was set to 100%, and individual group values were expressed as a percentage of this total. Heatmaps were then created with the “geom_tile” function in ggplot2 (version 3.5.1) [31].

## Results

### Overview of VOCs detected in the rhizosphere

In total, 12 VOCs were detected in the rhizosphere of apple plants grown in rhizoboxes (Table 2). Seven of the twelve VOCs were identified, while five remained unknown.

**Table 2:** List of rhizosphere VOCs and their characteristics detected in apple plant–soil rhizoboxes sampled with Sorb-Stars^®^

Symbol	Abbreviation	Full name	CAS	Formula	RT	RI_cal_	RI_lit_	Ions (*m/z*)	Molecular weight	Compound class
	FM	2-furanmethanol	98-00-0	C_5_H_6_O_2_	5.78	885	865	98_44_81_53_43	98	Other cyclic compound
	LF	longifolene	475-20-7	C_15_H_24_	18.17	1418	1409	91_161_79_93_94	204	Sesquiterpene
	HP	2-(2-hydroxypropoxy)-1-propanol	106-62-7	C_6_H_14_O_3_	9.6	1048	1034	59_45_44_103_91	134	Aliphatic compound
	DT	α-dihydroterpineol	498-81-7	C_10_H_20_O	12.07	1150	1132	59_55_81_43_44	156	Monoterpene
	TP	α-terpineol	98-55-5	C_10_H_18_O	13.2	1197	1195	59_93_81_43_121	154	Monoterpene
	BF	3(2H)-benzofuranone	7169-34-8	C_8_H_6_O_2_	13.63	1215	1207	134_105_76_50_77	134	Aromatic compound
	BH	butylated hydroxytoluene	128-37-0	C_15_H_24_O	20.28	1520	1497	205_57_44_220_55	220	Aromatic compound
	UNK1	NA	NA	NA	6.96	938	NA	70_42_71_43_44	NA	NA
	UNK2	NA	NA	NA	9.53	1045	NA	43_109_110_44_85	NA	NA
	UNK3	NA	NA	NA	9.59	1149	NA	43_44_144_101_72	NA	NA
	UNK4	NA	NA	NA	23.19	1672	NA	85_101_135_151_103	NA	NA
	UNK5	NA	NA	NA	25.66	1809	NA	161_176_44_55_91	NA	NA

Sorted by compound class, and in compound class by RT

(DB5-MS, 30 m when available; otherwise general value for non-polar columns; source: NIST 20 mass spectral library)

*RI*_*cal*_ calculated RI, *RI*_*lit*_ literature RI

Across the chromatograms used for volatile profiling, 96–98% of all peaks were contaminants, with the vast majority being siloxanes. This is highlighted in a representative chromatogram (Fig. S6), where numerous signals were present, but only a few corresponded to VOCs.

### VOC dynamics across trapping setups and time points

**Fig. 3:**
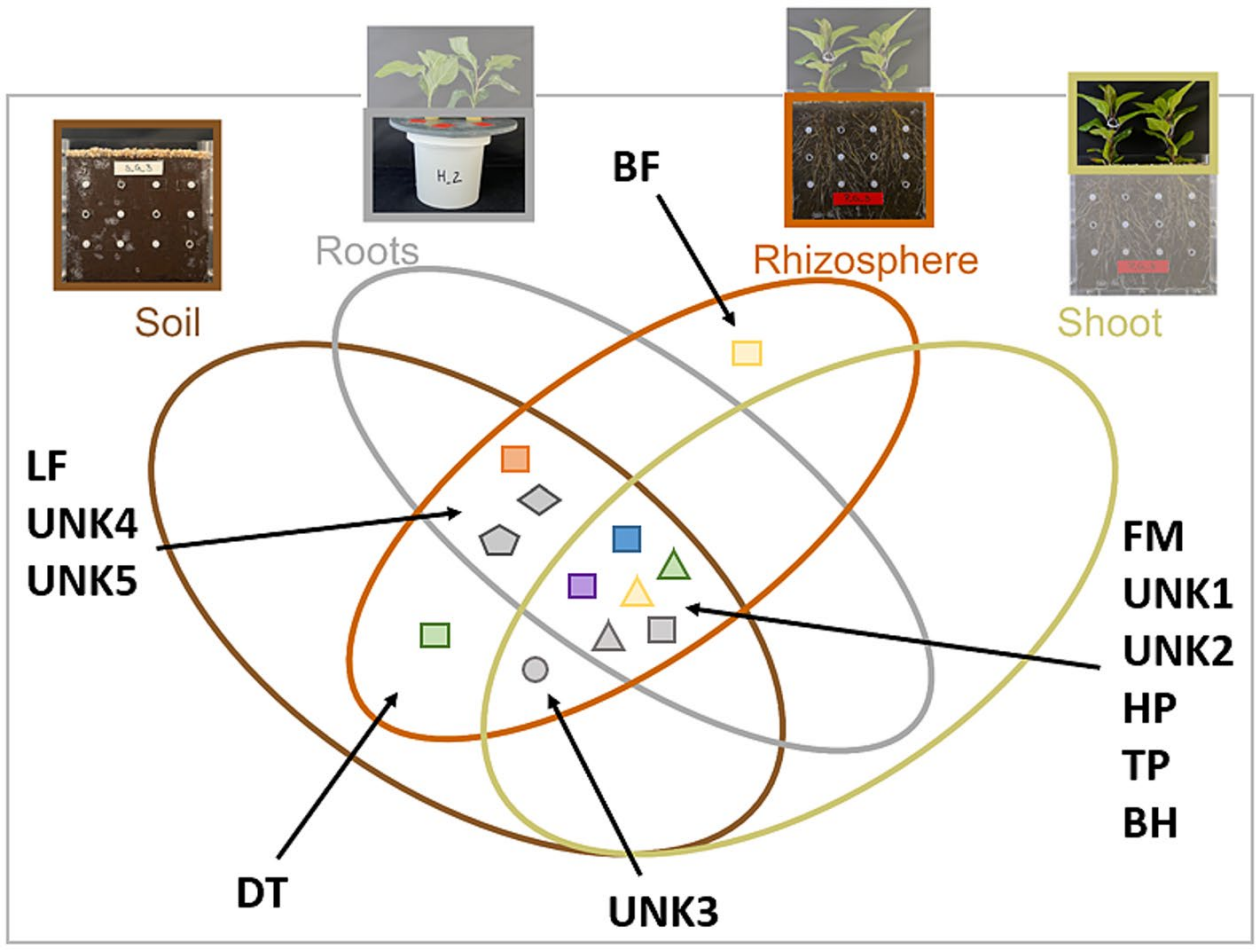
VENN diagram depicting the occurrence of VOCs detected in the rhizosphere across all sampled trapping setups. Each symbol corresponds to one individual VOC. The icons depicting the different trapping setups, which define the origins rhizosphere, soil, root, and shoot, indicate which parts of the experimental systems were sampled. Soil, root, and shoot samples were not searched for exclusive VOCs. Therefore, only their overlap with rhizosphere VOCs is depicted. Abbreviations denote the following VOCs: *BF* 3(2H)-benzofuranone, *BH* butylated hydroxytoluene, *DT* α-dihydroterpineol, *FM* 2-furanmethanol, *HP* 2-(2-hydroxypropoxy)-1-propanol, *LF* longifolene, *TP* α-terpineol

Six of the twelve detected VOCs were present across all origins, and three were found in all except the shoot (longifolene (LF), UNK4, UNK5; Fig. 3). Among the remaining VOCs, one was detected in all origins except roots (UNK3), one was found only in soil in addition to the rhizosphere (α-dihydroterpineol (DT)) and one appeared exclusively in the rhizosphere (3(2H)-benzofuranone (BF)).

**Fig. 4:**
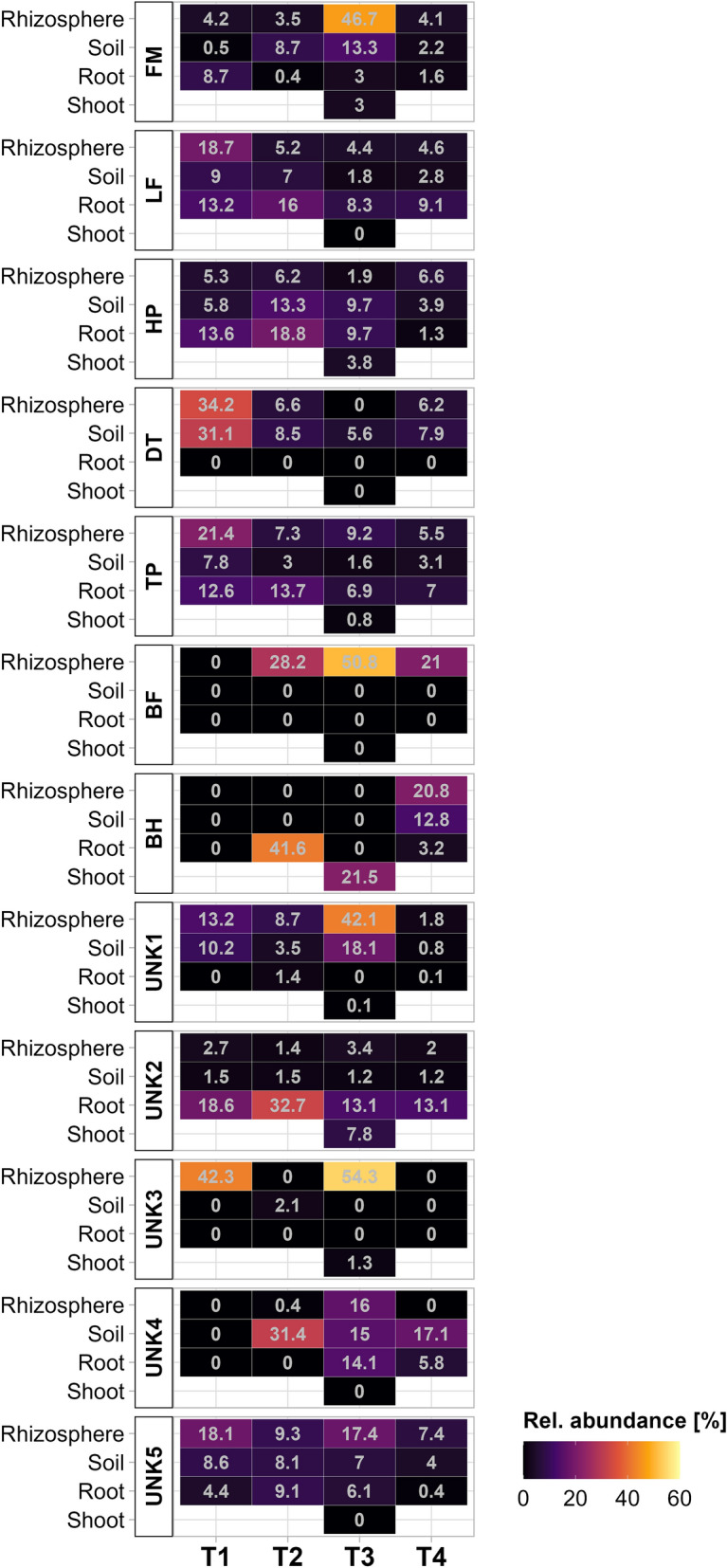
Heatmap showing the relative abundance of detected VOCs (%) grouped by sampling time (T) and trapping setups, which represent VOC origins (rhizosphere, root, soil, shoot). The color intensity indicates the relative abundance, ranging from dark purple (low abundance) to bright yellow (high abundance). The corresponding relative abundance values are displayed on the tiles. Abbreviations denote the following VOCs: *FM* 2-furanmethanol, *LF* longifolene, *HP* 2-(2-hydroxypropoxy)-1-propanol, *DT* α-dihydroterpineol, *TP* α-terpineol, *BF* 3(2H)-benzofuranone, *BH* butylated hydroxytoluene

The distribution patterns of individual VOCs were not uniform (Fig. 4). Depending on the compound, different origins and sampling times exhibited the highest relative abundance. Notably, for several VOCs (2-furanmethanol (FM), UNK1, UNK3, BF), the highest relative abundance (> 40%) was observed in the rhizosphere samples at time point 3. Other VOCs (UNK2, butylated hydroxytoluene (BH)) exhibited the highest relative abundance (> 30%) in root samples at the second time point. For 2-(2-hydroxypropoxy)-1-propanol (HP), no single time point or origin stood out, with all abundances remaining below 20%. A similar distribution can be observed for LF and UNK5, except that emittance from the shoot was absent. UNK4 was unique in that soil samples clearly dominated its profile, particularly at the second time point (> 30%). For DT, soil also showed a high relative abundance, but this VOC was present in the rhizosphere as well, with the combined contribution exceeding 60%.

## Discussion

### Methodological strengths and limitations

With our method, VOCs in the rhizosphere of soil-grown apple plants were successfully captured. This demonstrates that our experimental approach can be effectively applied to investigate how plant–soil interactions are shaped by VOCs.

But with only 12 detected VOCs, it can be questioned whether the full range of VOCs present in the rhizosphere was caught. VOCs may fail to reach the adsorbents due to their spatial distance from the emitter. This is particularly the case in soil-based systems, where gas diffusion can be limited. High pore water content reduces gas transport, and VOC dissolution in water can further restrict their mobility [32]. Additionally, depending on factors such as VOC concentration and polarity, clay content, and air humidity, VOCs can adsorb onto soil mineral surfaces [33] or soil organic matter [32]. Future studies could include plant species with well-characterized volatile profiles, such as maize (*Zea mays*) [34, 35], to validate VOC detection efficiency. In addition, the rhizobox experiment could be repeated using different adsorbents to systematically compare their performance.

With the described steps of volatile profiling, a clear separation between VOCs and contaminants was achieved. Most of the observed contaminant peaks likely originated from experimental materials (e.g., perforated microcentrifuge tubes and adsorbents), instrumental background signals (such as GC column bleed), or ambient air (e.g., within the growth chamber). Of these sources, adsorbent- and instrument-derived contaminants were effectively characterized using unexposed Sorb-Stars^®^ that were run alongside the samples during GC/MS. To identify the growth chamber-derived background, we placed an adsorbent in an empty rhizobox during the experiment. However, several peaks were detected that could be confidently annotated as plant- or microbe-derived VOCs. As empty rhizoboxes contain neither soil nor water that limit diffusion, they pick up any VOCs released into the growth chamber from the neighboring systems, in particular those produced from neighboring shoots. Consequently, the empty rhizoboxes present in the chamber during the experiment were unsuitable as “chamber blanks”. In future work, it may be useful to collect background samples before or after rhizobox setup.

The majority of the detected VOCs could be identified. All of them have been documented in the literature as characteristic VOCs in biological systems (Table 3), though their specific functions remain largely unexplored. To the best of our knowledge, this is the first report of their occurrence in apple. It should be noted that butylated hydroxytoluene (BH) and 2-(2-hydroxypropoxy)-1-propanol (HP) have not only been reported from biological sources but also in association with plastics [36, 37]. However, the absence of both compounds in the non-exposed “blank” adsorbents suggests a biological origin in our experiment.

**Table 3:** Literature report of the identified VOCs. See Table S1 for synonyms included in the literature search

Abbreviation	Full name	Origin^a^	Reference^b,c^	Detected with
FM	2-furanmethanol	Microbes	mVOC 4.0 database	Diverse prokaryotic and eukaryotic strains cultured in vitro
		Plants (belowground)	i.a., Abd El-Aty et al. [38]	*Panax ginseng* (solvent-free solid injection + GC/MS)
		Plants (aboveground)	i.a., Kim et al. [39]	Pepper *(Capsicum annuum)* fruits (solvent-free solid injection + GC/FID)
LF	Longifolene	Plants (belowground)	Jassbi et al. [40]	Sagebrush *(Artemisia tridentata)*, (SPME collection in the headspace of soil-inserted tube + GC/MS)
		Plants (aboveground)	i.a., Butkiene et al. [41]	Juniper *(Juniperus communis)*, (bark oil + GC/MS)
HP	2-(2-hydroxypropoxy)-1-propanol	Plants (aboveground)	i.a., Setyati et al. [42]	Orchid *(Dendrobium linearifolium)*, (methanol leaf extract + GC/MS)
DT	α-dihydroterpineol	Microbes	mVOC 4.0 database	Diverse eukaryotic strains cultured in vitro
		Plants (aboveground)	i.a., Mockutė et al. [43]	St. John’s wort (*Hypericum perforatum),* (essential oil of aerial parts + GC/MS)
TP	α-terpineol	Microbes	mVOC 4.0 database	Diverse prokaryotic and eukaryotic strains cultured in vitro
		Plants (belowground)	Lourenço et al. [44]	Yarrow *(Achillea millefolium)*, (essential oil of roots + GC/MS)
		Plants (aboveground)	i.a., Ye et al. [45]	Pine (*Pinus yunnanensis*) (powdered needles in a dynamic push–pull system + GC/MS)
BF	3(2H)-benzofuranone	Plants (aboveground)	i.a., Besada et al. [46]	Persimmon (*Diospyros kaki*), (powdered fruits, headspace SPME + GC/MS)
BH	butylated hydroxytoluene	Microbes	i.a., Gharbi et al. [47]	Fungal strains isolated from olives (headspace SPME + GC/MS)
		Plants (aboveground)	Bouftira et al. [48], and citations therein	Common ice plant (*Mesembryanthemum crystallinum)*, (leaf extract + HPLC)

*FID* Flame Ionization Detector

^a^Assigned origins indicate the targeted system, not necessarily the emitter. E.g., VOCs attributed to plants may be produced by plant-associated microbes

^b^Microbial emitters were evaluated using the mVOC database [49]. If no entries were available, further literature was searched

^c^“i.a.” denotes that additional literature exists for the listed VOC and origin

Some VOCs remained unidentified, primarily due to the incomplete nature of spectral libraries (e.g., [50]). Consequently, alternative approaches have been proposed that employ the mass spectra to elucidate the chemical structure of VOCs ([51], and citations therein). Incorporating these approaches in future studies may enable the identification of currently unknown compounds.

Furthermore, it should be noted that the aim of the developed method was to measure the volatile emissions as “naturally” as possible by avoiding any disturbances to the system. Although they are more representative of natural conditions than hydroponics, pot experiments are still artificial systems [52]. It is thus likely that the profiles of volatile emissions would differ under field conditions. For example, Kesselmeier et al. [53] found emissions of short-chain organic acids from several tree species to be distinctly lower under laboratory conditions. To adequately evaluate complex phenomena observable at field scale, sampling under field conditions is necessary to achieve a comprehensive understanding. Accordingly, this method should be further developed for field-scale application.

### Tracing the origin of the VOCs detected in the rhizosphere

With our multiple trapping set-ups, we were able to recognize six VOCs (2-furanmethanol (FM), UNK1, UNK2, 2-(2-hydroxypropoxy)-1-propanol (HP), α-terpineol (TP), butylated hydroxytoluene (BH)) as the “core volatilome,” which appear to be produced by numerous or highly abundant emitters. In contrast, the remaining VOCs exhibited a more setup-specific occurrence (Fig. 3). This aligns with Schenkel et al. [7], who differentiated core and organism-specific VOCs of bacteria and fungi, illustrating both overlap and specificity in volatile signals.

Two of the six core VOCs, FM and TP, are widely reported in microbial and plant volatile studies (Table 3), highlighting their broad distribution. For BH, we found no prior reports as a root-associated VOC, possibly reflecting research gaps given the limited focus on belowground plant VOCs. Similarly, to our knowledge, HP has not been reported as a root VOC. We also found no prior studies documenting microbial emission of HP. This contrasts with our own findings, which suggest that microbes can emit HP, as evidenced by its presence in the soil-only system.

Longifolene (LF)—together with UNK4 and UNK5—appears to be derived from both soil and plants, as these VOCs were detected in all but the shoot-only setup. Contrary to our results, no prior reports were found describing LF as a microbial VOC, but it is frequently reported as a compound in shoot volatile analyses (e.g., [41]).

Independent of these findings, LF, UNK4, and UNK5 demonstrate that root- and shoot-emitted VOCs were not identical. The same applies to UNK3, which appeared in the shoot but was absent from the root samples.

According to our results, α-dihydroterpineol (DT) appears to be emitted exclusively by soil microbes, as it was only detected in rhizosphere and soil samples. Contrary to these findings, DT has been reported from aboveground plant tissues (e.g., [43]) in addition to microbial sources (mVOC 4.0 database).

The VOC 3(2H)-Benzofuranone (BF) was the only one exclusively detected in rhizosphere samples, suggesting that its production depends on direct root-soil interaction. This was supported by Fig. 4, which shows that BF was detected only from the second sampling onward. Later samplings coincided with a higher root length, which likely intensified root-soil interactions. No reports on BF as a rhizosphere VOC were found, possibly because of the research gap in belowground VOCs. Some studies note BF as a shoot VOC (e.g., [46]), which limits its generalization as a rhizosphere-specific VOC.

Overall, it is important to note that our trapping setups were not fully enclosed, and gas exchange likely occurred. This may have resulted in VOCs being detected in setups where they were not actually produced. Despite efforts to minimize this—by sealing sampling holes with aluminum foil and enclosing shoots in bags during sampling—some exchange was likely unavoidable. Additionally, distinguishing between plant-derived and microbial VOCs is challenging. Even in plant-only experimental systems, microbial volatile emissions may occur due to their ubiquitous presence. Without sterile conditions, VOCs specified as “plant-derived” cannot be attributed solely to plant emissions but may also originate from the plant-associated microbiome.

### Temporal variation of VOC release across trapping setups

The heatmap (Fig. 4) illustrates the specific distribution patterns of volatile abundances, demonstrating that different origins and time points were particularly pronounced for each VOC. Similar tendencies were observed for only a few compounds.

The highest relative abundances of FM, UNK1, and UNK3 were observed in the rhizosphere samples at time point 3. This third sampling was notable as it coincided with visible signs of a mildew infection (Fig S7). Mildew can alter plant-associated microbial communities [54] and elicit various plant responses [55], both of which may contribute to shifts in volatile emissions. This was supported by the work of Wu et al. [56], who demonstrated that the volatile profile of fermented tobacco leaves infected with mildew was significantly different from that of uninfected leaves. However, if the observed increases were solely due to mildew infection, it is unclear why this effect was limited to rhizosphere samples and not evident in the shoots, where mildew symptoms occurred. Similarly, an effect on the root samples would be expected, given that the hydroponically grown plants also exhibited disease symptoms. Moreover, the absence of an effect at the fourth sampling, despite the infection still being visible, is unclear.

At the second sampling, an increase of UNK2 and BH was observed in root samples. While hydroponic conditions may promote higher volatile levels due to unlimited diffusion, the restriction of this effect to two VOCs and one sampling remains unresolved.

Overall, the observed distribution patterns were complex and difficult to interpret, with no consistent period of highest volatile emission.

Two possible explanations may account for the absence of clear trends. First, consistent trends may indeed not exist. As VOC release is regulated by numerous interacting factors that can change rapidly [4], emissions might be too dynamic to exhibit consistent patterns. Second, these trends were not captured with our experimental approach for reasons that remain unclear. Dudareva et al. [57] reported that volatile emissions are developmentally regulated, suggesting well-defined temporal patterns. A key limitation could be that volatile abundances may not be directly comparable across systems. Plant growth varied significantly between hydroponic and rhizobox systems, with lower root but higher shoot growth in hydroponics (Fig. S8), which could shift the timing of certain physiological processes. Moreover, differences in diffusion dynamics between soil and hydroponic setups, as well as uncertainty regarding the distance between emitters and adsorbents, may have affected VOC capture, thereby complicating direct comparisons further. In addition, the restriction to twelve VOCs may have limited our ability to detect the dominant patterns in volatile abundance.

## Conclusions

This paper presents a novel static sampling method for the in situ collection of VOCs in undisturbed plant–soil systems, enabling the capture of VOCs in the rhizosphere. With the described workflow, we identified about half of the VOCs, all of which corresponded to previously reported volatiles in biological systems. Using complementary trapping setups targeting roots, shoots, and soil, the detected VOCs could be classified into the “core volatilome” (present in all setups) and more setup-specific VOCs. An optimal time frame for sampling could not be determined, as the sampling time with the highest relative abundance varied among VOCs. Overall, the proposed method provides a powerful approach for investigating the largely unknown dynamics of rhizosphere VOCs. This work thus serves as a solid basis for future research on plant responses to biotic and abiotic stresses, plant adaptation to environmental conditions, and the inter- and intraspecific communication of rhizosphere microbial communities.

## Supplementary Information

Supplementary Material 1.

Supplementary Material 2.

Supplementary Material 3.

Supplementary Material 4.

Supplementary Material 5.

Supplementary Material 6.

Supplementary Material 7.

Supplementary Material 8.

Supplementary Material 9.

Supplementary Material 10.

## Data Availability

All data generated or analysed during this study are included in this published article and its supplementary information files.
