# Supplementary figures and images for: An efficient static sampling method for in situ measurement of rhizosphere volatile organic compounds in plant–soil systems
Source: Plant Methods. 2026 Jul 27;22:63. doi: 10.1186/s13007-026-01573-y (PMC13412312; doi:10.1186/s13007-026-01573-y)

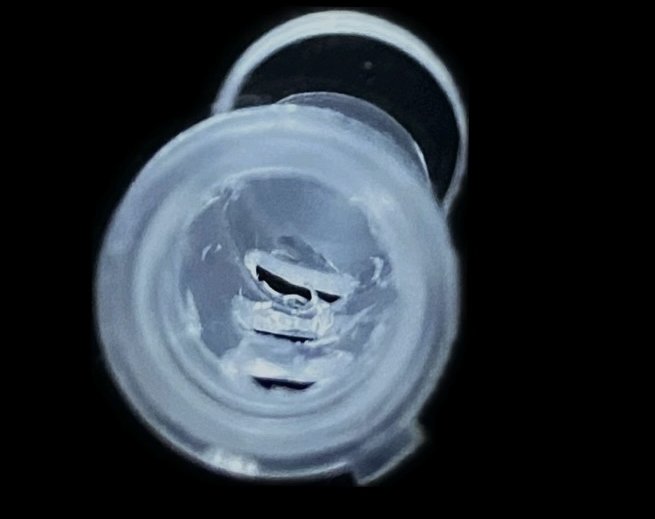

Supplement: Supplementary file 1 — Supplementary Material 1. [file 13007_2026_1573_MOESM1_ESM.jpg]

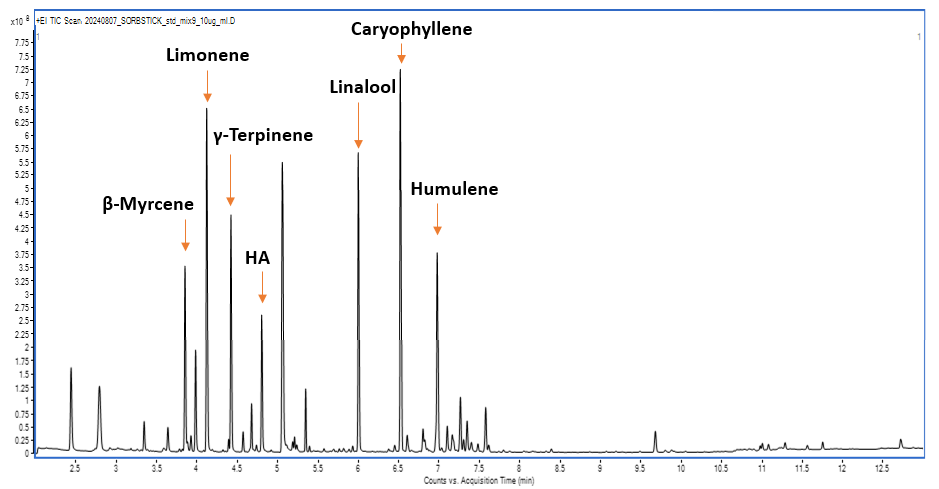

Supplement: Supplementary file 2 — Supplementary Material 2. [file 13007_2026_1573_MOESM2_ESM.png]

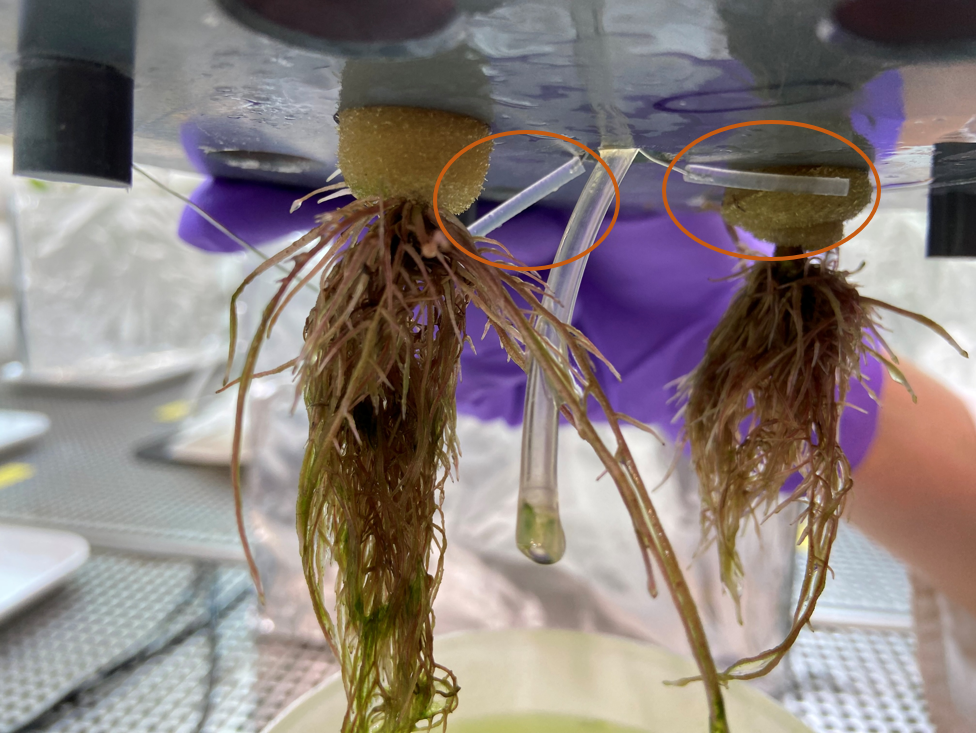

Supplement: Supplementary file 3 — Supplementary Material 3. [file 13007_2026_1573_MOESM3_ESM.png]

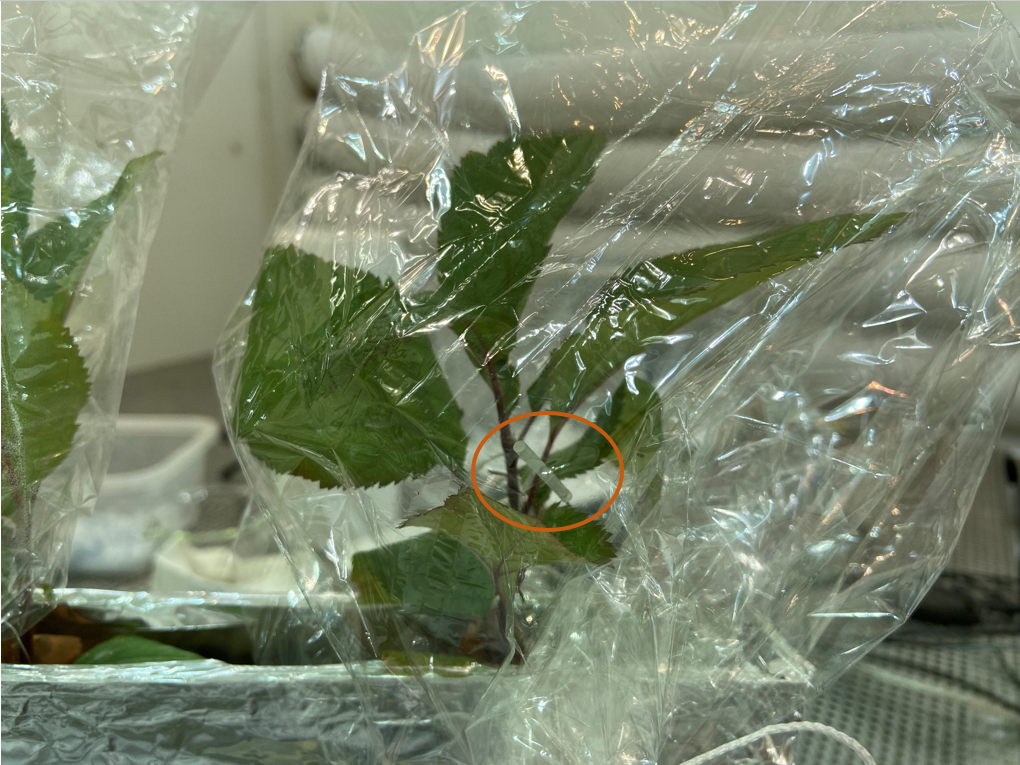

Supplement: Supplementary file 4 — Supplementary Material 4. [file 13007_2026_1573_MOESM4_ESM.png]

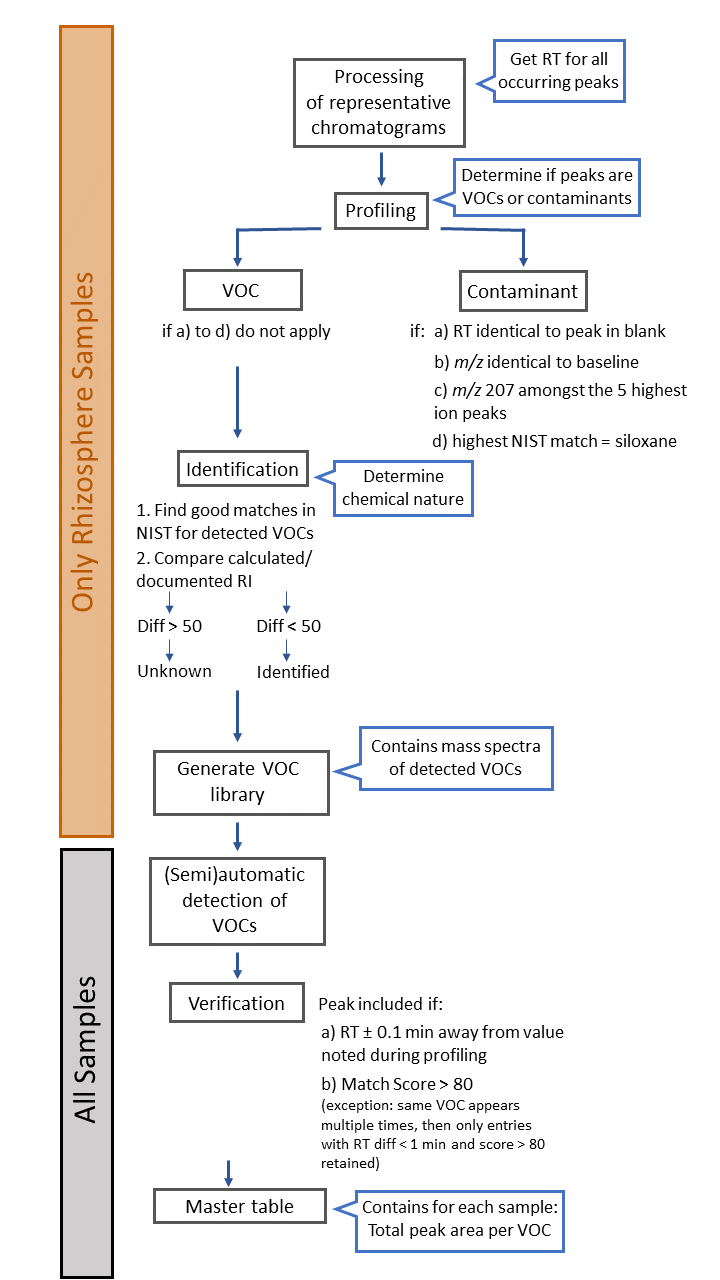

Supplement: Supplementary file 5 — Supplementary Material 5. [file 13007_2026_1573_MOESM5_ESM.png]

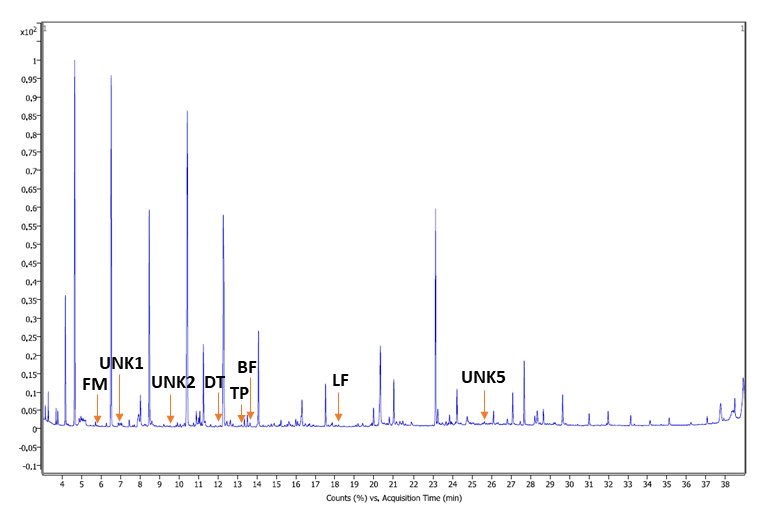

Supplement: Supplementary file 6 — Supplementary Material 6. [file 13007_2026_1573_MOESM6_ESM.png]

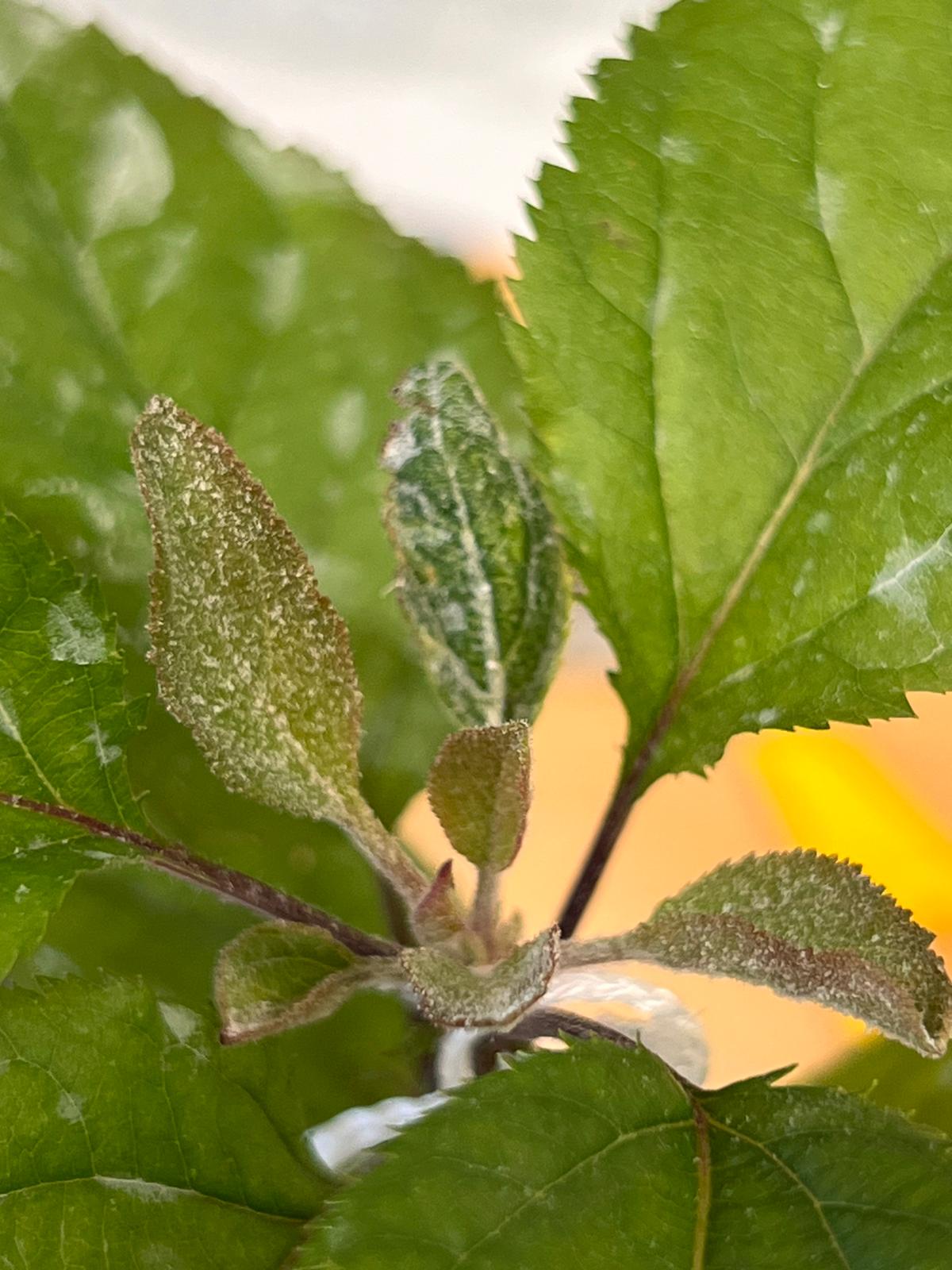

Supplement: Supplementary file 7 — Supplementary Material 7. [file 13007_2026_1573_MOESM7_ESM.jpeg]

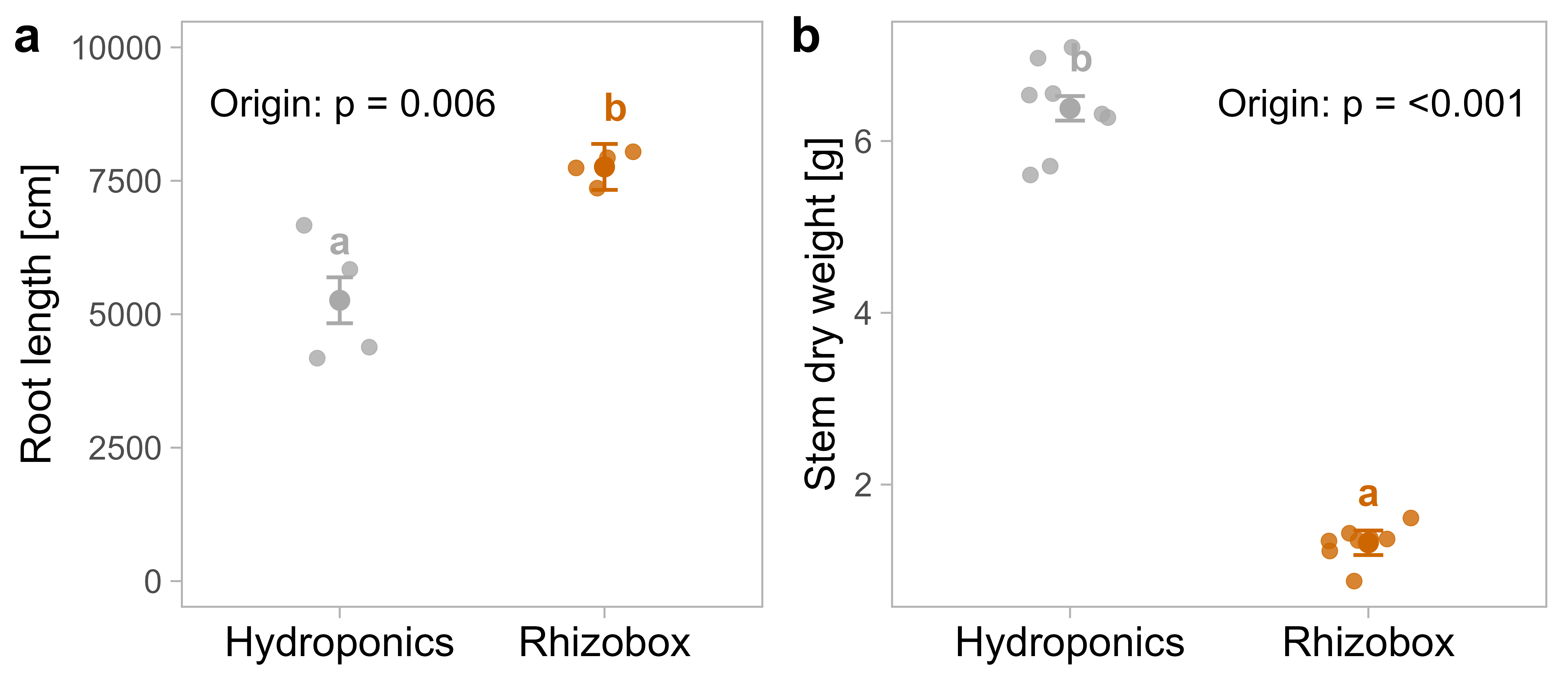

Supplement: Supplementary file 8 — Supplementary Material 8. [file 13007_2026_1573_MOESM8_ESM.png]
