# Supplementary material for: An efficient static sampling method for in situ measurement of rhizosphere volatile organic compounds in plant–soil systems
Source: Plant Methods. 2026 Jul 27;22:63. doi: 10.1186/s13007-026-01573-y (PMC13412312; doi:10.1186/s13007-026-01573-y)
Supplement: Supplementary file 9 — Supplementary Material 9. [file 13007_2026_1573_MOESM9_ESM.pdf]

Table S1. Identified VOCs and corresponding synonyms used for literature search.

Name abbreviated	Name full	Synonyms
FM	2-furanmethanol	furfuryl alcohol<br>α-furylcarbinol<br>2-(hydroxymethyl)furan
LF	longifolene	junipene<br>kuromatsuen
HP	2-(2-hydroxypropoxy)-1-propanol	1-propanol, 2-(2-hydroxypropoxy)-
DT	α-dihydroterpineol	cyclohexanemethanol, α,α,4-trimethyl-
TP	α-terpineol	3-cyclohexene-1-methanol, α,α4-trimethyl-<br>p-menth-1-en-8-ol
BF	3(2H)-benzofuranone	coumaran-3-one
BH	butylated hydroxytoluene	phenol, 2,6-bis(1,1-dimethylethyl)-4-methyl-<br>p-cresol, 2,6-di-tert-butyl-
